# Current knowledge of *Schisandra chinensis* (Turcz.) Baill. (Chinese magnolia vine) as a medicinal plant species: a review on the bioactive components, pharmacological properties, analytical and biotechnological studies

**DOI:** 10.1007/s11101-016-9470-4

**Published:** 2016-05-12

**Authors:** Agnieszka Szopa, Radosław Ekiert, Halina Ekiert

**Affiliations:** 1 Chair and Department of Pharmaceutical Botany, Jagiellonian University, Collegium Medicum, ul. Medyczna 9, 30-688 Kraków, Poland; 2 „Herbapol” Krakow S.A., ul Chałupnika 14, 31-464 Kraków, Poland

**Keywords:** Schizandra, Dibenzocyclooctadiene lignans, Schisandrin, Gomisin A, Triterpenoids, In vitro cultures

## Abstract

*Schisandra chinensis* Turcz. (Baill.) is a plant species whose fruits have been well known in Far Eastern medicine for a long time. However, schisandra seems to be a plant still underestimated in contemporary therapy still in the countries of East Asia. The article presents latest available information on the chemical composition of this plant species. Special attention is given to dibenzo cyclooctadiene lignans. In addition, recent studies of the biological activity of dibenzocyclooctadiene lignans and schisandra fruit extracts are recapitulated. The paper gives a short resume of their beneficial effects in biological systems in vitro, in animals, and in humans, thus underlining their medicinal potential. The cosmetic properties are depicted, too. The analytical methods used for assaying schisandra lignans in the scientific studies and also in industry are also presented. Moreover, special attention is given to the information on the latest biotechnological studies of this plant species. The intention of this review is to contribute to a better understanding of the huge potential of the pharmacological relevance of *S. chinensis.*

## Introduction

*Schisandra chinensis* (Turcz.) Baill.—Chinese magnolia vine; schisandra (eng.), schizandre de Chine (fr.), chinesische Beerentraube, chinesisches Spaltkölbchen (germ.), Лимoнник китaйcкий (rus.), wuweizi, 五味子 (chin.), gomishi, ゴミシ (jap.), 오미자, omija (kor.) is a plant species well-known in Traditional Chinese Medicine (TCM) and also in modern Chinese medicine. The first description of *S. chinensis* species can be found in a 1596 work on ancient Chinese medicine written by Li Shih-Chen—“Pên T’shao Kang Mu”. The fruit of the Chinese magnolia vine was used in the treatment of diseases of the gastrointestinal tract, respiratory failure, cardiovascular diseases, in the states of body fatigue and weakness, excessive sweating and insomnia. The material is also known from the traditional Russian medicine, in which it was described as a tonic, reducing hunger, fatigue, delaying the aging process, increasing vitality, and improving mental health (Fil’kin 1952; Bensky and Gamble 1993).

Recent researches on the biological activity of extracts from the fruit, as well as the main active components isolated from this raw material—dibenzo[a,c]cyclooctadiene lignans, have confirmed these properties and added new aspects relating to the action of *S. chinensis*.

The most valuable, collective sources of information on the latest research on this species are periodically appearing review publications. The first of the modern reviews from 1999 by Hancke et al. (1999) signalled different aspects of the pharmacology of *S. chinensis* fruit, focusing in particular on the antihepatotoxic, antioxidant and antitumoral activities, and on the effects on physical performance and on the central nervous system proved by scientific researches.

A very valuable study is the publication by Opletal et al. from 2004 (2004), in which the authors focused on dibenzocyclooctadiene lignans. They presented different methods of isolating these compounds and their biological activity, paying special attention to analytical methods. Another extensive study dealing with the issue of the pharmacology of fruit extracts of *S. chinensis* in Russian research and medicine is a publication from 2008 written by Panossian and Wikman (2008). This work raises the aspects of ethnobotany and the chemical composition and pharmacology of *S. chinensis* fruit. There are some review articles in national languages, for example in Polish, focused on different aspects of value of *S. chinensis* (Ekiert 2005; Szopa et al. 2012). In recent years there have also been reports on the biotechnological research of this species. The progress achieved in this area was presented in a monograph by our team in the form of a book chapter by Szopa and Ekiert (2014).

The objective of present paper is to systematize the latest reports on the chemical composition, biological activity and analytical testing of dibenzocyclooctadiene lignans and *S. chinensis* fruit extracts. It also includes a presentation of the position of *Schisandrae chinensis fructus* in the world pharmacopoeial documents. Moreover, attention is drawn to biotechnological studies on *S. chinensis*.

## Botanical background and occurrence

The genus Schisandra, according to various authors, includes from 20 to 30 species (Panossian and Wikman 2008; World Health Organization 2007). With the exception of one, all of the species have their natural habitats in East and South-East Asia, twelve of which are endemic to China (Wu et al. 2008). Only *S. glabra* (*S. coccinea*) can be found in natural habitats in the south-eastern part of North America (Foster 1998; Saunders 2000). The species of Schisandra differ from one another in small details in their morphological structure and the chemical composition of the leaves, stems, flowers, or fruits.

*Schisandra chinensis* Turcz. (Baill.)—Chinese magnolia vine, is a dioecious climbing plant. It has its natural habitats in north-eastern China, Korea, Japan, in the eastern part of Russia, in Primorsk, on the Kuril Islands and in the south of the Sakhalin Island (Bensky and Gamble 1993). The plant requires conditions of moderate humidity and light, together with a wet, humus-rich soil. It occurs in mixed forests, especially on the periphery, also by streams and brooks, usually on sandy soils (Genders 1977). The species is grown mainly in China, Korea, Russia, and also in North America and in many European countries, especially for ornamental purposes.

As is specified below, the medicinal raw material “Wuweizi (Beiwuweizi)” consists of the fruits of *S. chinensis*, “Nanwuweizi” refers to the fruits of another Schisandra species—*S. sphenanthera*, which is common in southern China provinces (Lu and Chen 2009; Opletal et al. 2004). These fruits of two Schisandra species look alike and can only be distinguished by microscopic examination—the fruit of *S. sphenanthera* has numerous oil cells and crystals in the form of prisms or rotundish aggregates in the mesocarp. These diagnostic elements are not present in the fruit of *S. chinensis* (Upton and Petron 1999; Upton et al. 2011). In recent years, many analytical tests have also identified significant differences in the chemical composition of the fruit of these two species (Lu and Chen 2009).

The fruit of *S. chinensis* are berries, about 1 cm in diameter, whose exocarp and mesocarp are smooth, shiny, of a strong red colour. The berries are clustered in grape-like bunches about 10 cm long; in Europe, they ripen in late August and early September. There are 1–2 kidney-shaped seeds inside each berry (Upton and Petron 1999; Upton et al. 2011; World Health Organization 2007; Wu et al. 2008).

## Position in worldwide pharmacopoeial documents

The medicinal raw material harvested from *S. chinensis* according to traditional applications as well as current information in modern specification documents are the fruits. The fruits of *S. chinensis* have long had their pharmacopoeial monographs in the Chinese (2005), Japanese (2006), Korean (2002) as well as Russian Pharmacopoeias (1990). The knowledge on the fruits of *S. chinensis* reached the European and American countries relatively recently. The first monograph on Schisandrae fruit can be found in The American Pharmacopoeia from 1999 (1999). The first official, internationally recognized, monograph on this raw material has been available only since 2007 in the International Pharmacopoeia edited by WHO (2007).

In the European documents, the fruits of the Chinese magnolia vine have been listed as a pharmacopoeial raw material for only a few years—since 2008. A monograph of the fruit of this species, under the name of *Schisandrae chinensis fructus* appeared for the first time in Supplement 6.3 to the 6th edition of the European Pharmacopoeia (2008). In the latest 8th edition of the European Pharmacopoeia (2013) valid since 2014, the monograph on *Schisandrae chinensis fructus* is still in its unchanged form. Nevertheless, the description of the material in these documents always distinguishes it from another, also popular, South China species—*S. sphenanthera*.

Nowadays, despite its presence in the current pharmacopoeial documents, *S. chinensis* is still an interesting object in the field of medicinal chemistry and drug discovery (Hancke et al. 1999; Opletal et al. 2004; Panossian and Wikman 2008), and has come to the foreground of interest of phytochemical and pharmacological research mainly due to the bioactive dibenzocyclooctadiene lignans (Shi et al. 2014b).

## Characteristic and current data on chemical composition

### *Schisandra chinensis*

The *S. chinensis* fruit is characterized by a rich, still not fully known, chemical composition. The most important components of the berries are dibenzo[a,c]cyclooctadiene lignans (Table 1). Because their occurrence is limited to this particular species, these compounds are often referred too, even in professional publications, as “schisandra lignans” (Hancke et al. 1999; World Health Organization 2007).

**Table 1:** The chemical parameters of selected dibenzocyclooctadiene lignans (Chiu et al. 2008; He et al. 1997; Deng et al. 2008; Wang et al. 2011)

Lignan name	Molecular formula	Configuration	Molecular weight (g/mol)	Fragment ions pattern	Chemical structure
Deoxyschisandrin	C_24_H_32_O_6_	R-biphenyl	416	439(M+Na)^+^, 417(M+H)^+^, 402, 386(M+H-CH_3_O)^+^, 371, 370, 347(M+H-C_5_H_10_)^+^, 332, 331, 316, 315, 301, 285, 236, 182	
Gomisin A	C_23_H_28_O_7_	R-biphenyl	416	439(M+Na)^+^, 417(M+H)^+^, 399(M+H-H_2_O)^+^, 384, 369, 368(M+H-H_2_O-CH_3_O)^+^, 357, 353, 343, 337, 323	
Schisandrin	C_24_H_32_O_7_	R-biphenyl	432	455(M+Na)^+^, 433(M+H)^+^, 415(M+H-H_2_O)^+^, 400, 384(M+H-H_2_O-CH_3_O)^+^, 373, 369, 359, 353, 338, 322	
Schisandrin B	C_23_H_28_O_6_	R-biphenyl	400	423(M+Na)^+^, 418(M+NH_4_)^+^, 401(M+H)^+^, 386, 371(M+H-CH_3_O)^+^, 331(M+H-C_5_H_10_)^+^, 300(M+H-C_5_H_10_-CH_3_O)^+^, 285, 270, 258, 242, 227	
Schisandrin C	C_23_H_24_O_6_	S-biphenyl	384	407(M+Na)^+^, 385(M+H)^+^, 354(M+H-CH_3_O)^+^	
γ-Schisandrin	C_23_H_28_O_6_	R-biphenyl	400	423(M+Na)^+^, 418(M+NH_4_)^+^, 401(M+H)^+^, 386, 371(M+H-CH_3_O)^+^, 331(M+H-C_5_H_10_)^+^, 300(M+H-C_5_H_10_-CH_3_O)^+^, 285, 270, 258, 242, 227	
Schisanthenol	C_23_H_30_O_6_	R-biphenyl	402	425(M+Na)^+^, 403(M+H)^+^, 385(M+H-H_2_O)^+^, 371, 370(M+H-H_2_O-CH_3_)^+^, 357, 346, 339(M+H-H_2_O-CH_3_-CH_3_O)^+^, 222, 182	
Schisantherin A	C_30_H_32_O_9_	S-biphenyl	536	575(M+K)^+^, 559(M+Na)^+^, 554(M+NH_4_)^+^, 537(M+H)^+^, 415(M+H-C-_7_H_6_O_2_)^+^, 397(M+H-C_7_H_6_O_2_-H_2_O)^+^, 385, 373, 371(M+H-C_7_H_6_O_2_-C_2_H_4_O)^+^, 356, 344, 343,341, 340(M+H-C_7_H_6_O_2_-C_2_H_4_O-CH_3_O)^+^, 123, 106, 78	
Schisantherin B	C_28_H_34_O_9_	S-biphenyl	514	537(M+Na)^+^, 532(M+NH4)^+^, 515(M+H)^+^, 415(M+H-C_5_H_8_O_2_)^+^, 397(M+H-C_5_H_8_O_2_-H_2_O)^+^, 385, 373, 371(M+H-C_5_H_8_O_2_-C_2_H_4_O)^+^, 356, 344, 341, 340, 101, 84, 56	

The amounts of dibenzocyclooctadiene lignans in the fruits of *S. chinensis* are influenced by the degree of fruit maturity, harvest time, the habitat and its location. These amounts vary over a wide range, from 4 to 19 g % DW (European Pharmacopoeia 8.0 2013; Opletal et al. 2004; World Health Organization 2007). Data from 2007, included in the WHO monograph, referred to the presence in the fruits of *S. chinensis* of thirty compounds from this group. The earliest dibenzocyclooctadiene lignans to be identified, and found to occur in the largest quantities in the fruits, were: schisandrin, schisandrins B and C, γ-schisandrin, schisantherins A and B, schisanthenol, deoxyschisandrin, gomisins A and G. These compounds have a number of synonymous names that are collected in Table 2 (Ikeya et al. 1979; Kochetkov et al. 1961; Opletal et al. 2004; World Health Organization 2007). The latest studies report on newly identified structures from this group of lignans, which have been given the name schisanchinins A–D (Hu et al. 2014), and the first example of naturally occurring *N*-containing lignan featuring a nicotinoyl group—nicotinoylgomisin (Shi et al. 2014b). In addition, other types of lignans have been isolated from the fruits of *S. chinensis*—dibenzylbutanes lignans: schineolignans A–C, and tetrahydrofuran lignans: schinlignins A and B (Xue et al. 2010; Zhang et al. 2014b) (Table 3).

**Table 2:** The main dibenzocyclooctadiene lignans and their synonymous names

Dibenzocyclooctadiene lignans	Synonymous names
Deoxyschisandrin	Deoxyschizandrin, Dimethylgomisin J, Schisandrin A, Schizandrin A, Wuweizisu A
Gomisin A	Schisandrol B, Schizandrol B, Wuweizisu B, Wuweizichun B, Wuweizi alcohol B
Schisandrin	Schizandrin, Schisandrol A, Schizandrol A, Wuweizichun A
Schisandrin B	(−)Schizandrin B, Gomisin N
Schisandrin C	Schizandrin C, Wuweizisu C
γ-Schisandrin	(±)γ-Schizandrin B, Wuweizisu B
Schisanthenol	Schizantherol, (+)-Gomisin K3
Schisantherin A	Gomisin C, Schizandrer A, Wuweizi ester A
Schisantherin B	Gomisin B, Schizandrer B, Wuweizi ester B

**Table 3:** New confirmed lignans of *S. chinensis* and *S. sphenanthera*

*S. chinensis*		*S. sphenathera*	
Compound	References	Compound	References
Dibenzocyclooctadiene lignans			
Schisanchinins A–D Nicotinoylgomisin	Shi et al. (2014b)	Methylgomisin O Chloromethyl Schisantherin B Schisphenlignans A–E	Ren et al. (2010)
Polyoxygenated C18-dibenzocyclooctadiene lignans			
nd		Arisanschinins M and N Schisphenin A	Chen et al. (2013b)
Dibenzylbutanes lignans			
Schineolignans A–C	Xue et al. (2010)	nd	
Tetrahydrofuran lignans			
Schinlignins A and B	Xue et al. (2010)	nd	
2,5-Diaryltetrahydrofuran lignans			
nd		Chicanine Epigalbacine Ganschisandrine	Liang et al. (2013)
4-Aryltetralin lignans			
nd		Schisandrone	Liang et al. (2013)
2,3-Dimethyl-1,4-diarylbutane lignans			
nd		Anwulignan Sphenanlignan	Liang et al. (2013)

nd - not detected

**Fig. 1:**
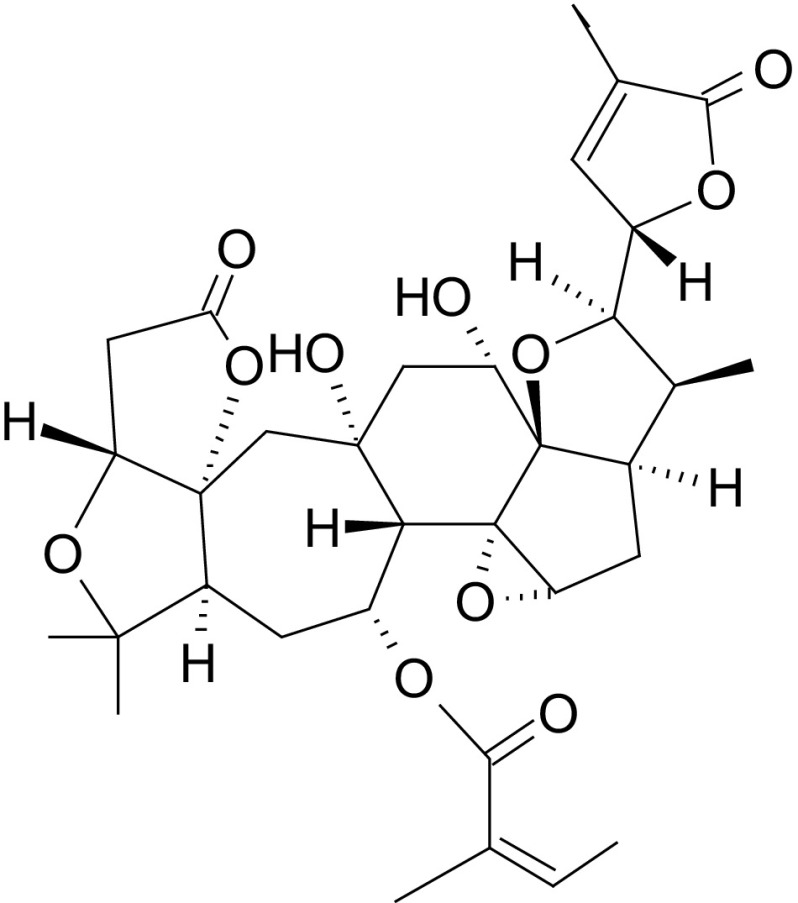
Chemical structure of Wuweizidilactone I acc. to Xue et al. (2010)

Analyses of the composition of the fruits have in recent years been focused on finding new biologically active compounds. The berries of *S. chinensis* have been found to contain compounds identified as belonging to the group of triterpenoids, such as: pre-schisanartanes—schisanartanins A and B, and 3,4-seco-21,26-olide-artane triterpenoid—wuweizilactone acid (Huang et al. 2007; 2008; Xia et al. 2015; Xue et al. 2010). Another interesting, recently isolated, structure is a compound from the group of 18-norschiartanes known as bisnortriterpenoids, which do not have C-18 and C-28; it is a highly oxygenated nortriterpenoid with unusual skeletons—wuweizidilactone I (Xue et al. 2010) (Fig. 1).

**Fig. 2:**
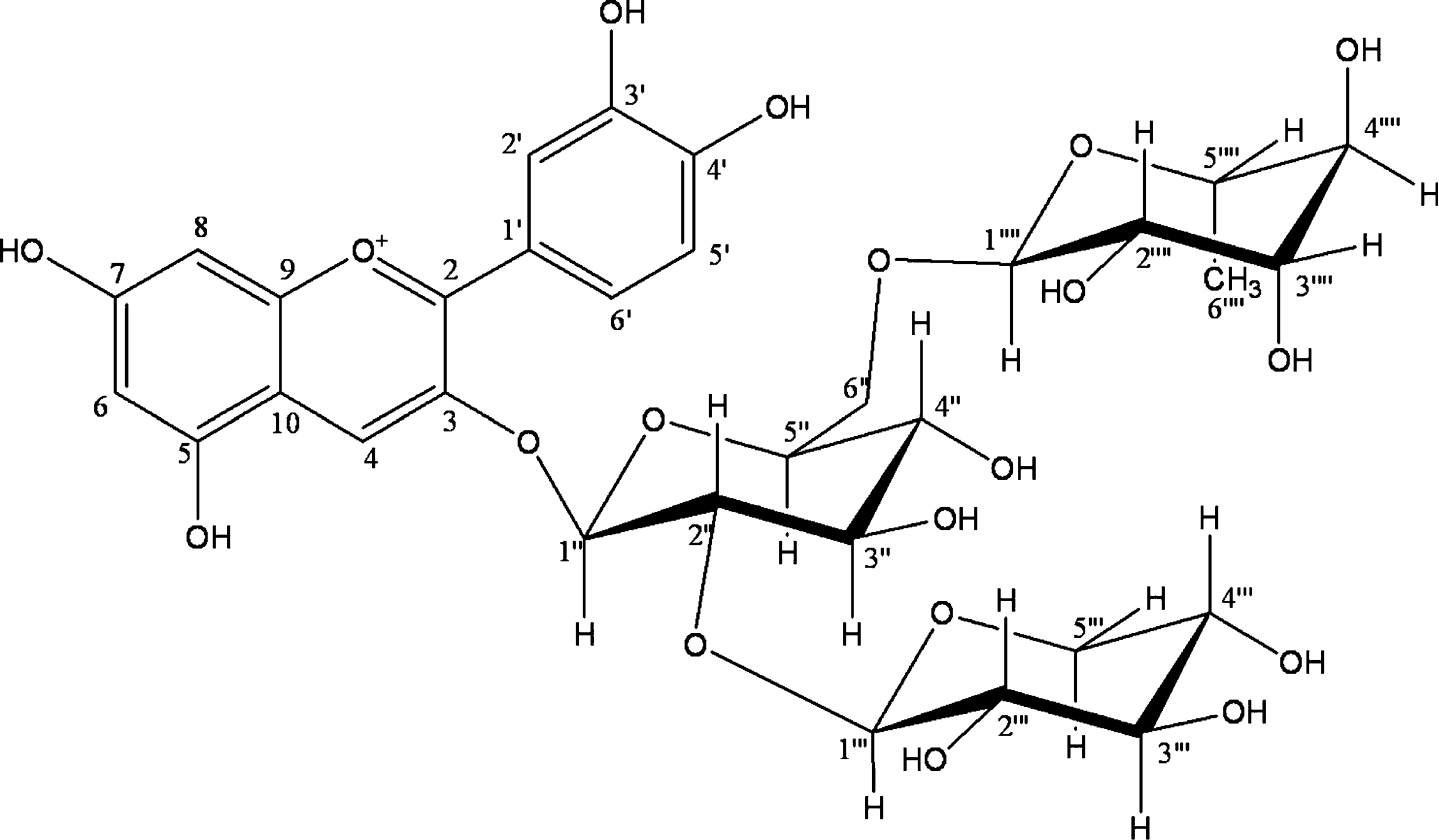
The structure of predominant colourant of *S. chinensis* fruit—Cyanidin-3-O-xylosylrutinoside (Cya-3-O-xylrut) acc. to Kim et al. (2009)

A few years ago, the compound considered to be the predominant colourant of *S. chinensis* fruit was identified as cyanidin-3-O-xylosylrutinoside (Cya-3-O-xylrut) (Kim et al. 2009; Ma et al. 2012a) (Fig. 2).

The fruits of *S. chinensis* also contain an essential oil in an amount of about 3 %. Sesquiterpenes are dominant in the essential oil. Oxygenated monoterpenes, monoterpenes and oxygenated sesquiterpenes are present in smaller quantities (about 5 %). Ylangene, β-himachalene, α-bergamotene and β-chamigrene are the main components, comprising about 75 % of the oil (Chen et al. 2011).

The most recent studies have also included analyses of the composition of different groups of polyphenols. Among the phenolic acids found in the fruits, our team has confirmed the presence of: chlorogenic, p-coumaric, p-hydroxybenzoic, protocatechuic, salicylic and syringic acids (Szopa and Ekiert 2012). Other authors have additionally proved the presence of gentisic acid (Mocan et al. 2014). The same team also confirmed the presence of compounds from the group of flavonoids: hyperoside, isoquercitrin, rutin and quercetin (Mocan et al. 2014).

Moreover, *S. chinensis* fruits are rich in polysaccharides and monosaccharides (glucose, fructose, arabinose and galactose), vitamins (C and E), phytosterols (stigmasterol, β-sitosterol), organic acids (citric, malic, fumaric and tartaric acids) and bioelements (Ca, Mg, Fe, Mn, B, Zn, Cr, Ni, Cu, Co) (Hancke et al. 1999; Szopa and Ekiert 2014; Tong et al. 2012).

**Fig. 3:**
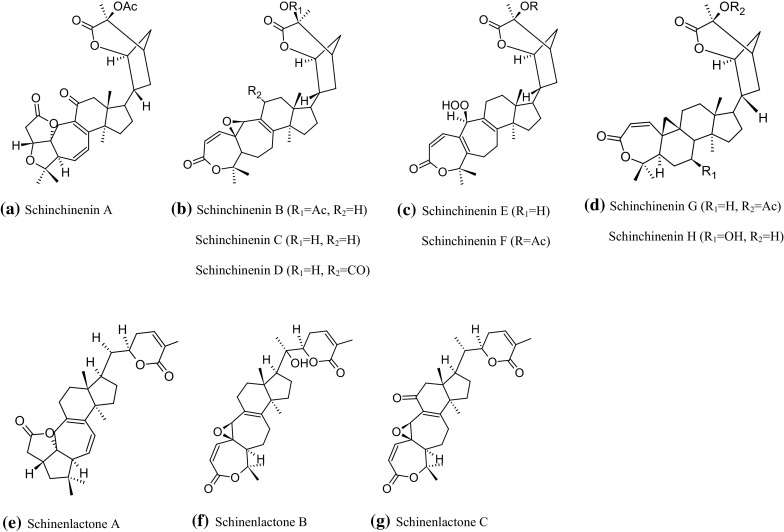
Examples of recently identified triterpenoids from *S. chinensis*: **a**–**d** Schinchinenins A–H, **e**–**g** Schinenlactones A–C, acc. to Song et al. (2013)

Apart from the fruits, the chemical composition of the leaves, and often of the stems with the leaves of *S. chinensis*, is also analyzed. On the basis of the examinations of the results of analyses of the chemical composition of the green parts of *S. chinensis*, particularly noteworthy is the large number of compounds from the group of terpenoids, especially distinctive terpenoids from cycloartane groups, such as: schinchinenins A–H and schinchinenlactones A–C (Fig. 3). These compounds are highly oxygenated triterpenoids, which is quite rare among the constituents isolated from the Schisandra species (Song et al. 2013). Two schinesdilactone-type nortriterpenoids have also been identified: schinesdilactones A and B. Moreover, 16,17-seco-preschisanartane nortriterpenoids: schisdilactones A–G (Wang et al. 2012), were the first examples of these classes isolated from the leaves and stems of *S. chinensis*. Additionally, the first to be extracted from the leaves and stems of *S. chinensis* were also other triterpenoids: isoschicagenin C (Wang et al. 2012) and schicagenins A–C (Shi et al. 2014b).

Stems with the leaves of *S. chinensis* are also a source of dibenzocyclooctadiene lignans, e.g. schisandrin, gomisins A and J, pregomisin, angeloylgomisin H and Q (Zhu et al. 2015). In extracts from the leaves, apart from schisandrin and gomisin A, other compounds from this group have also been detected e.g. deoxyschisandrin, schisandrin B, gomisin G and schisantherin (Szopa and Ekiert 2011, 2013, 2015; Szopa et al. 2016a; Xiao et al. 2007), in significant but smaller quantities than in the fruits.

Apart from the above-mentioned compounds, the leaves have also been found to contain isoquercitrin as the main flavonoid, followed by hyperoside, rutin, myricetin, quercitrin, quercetin and kaempferol (Mocan et al. 2014); besides these, other flavonoid glycosides occur in the leaves, such as: ((+)-isoscoparine and quercetin 3-O-β-L-rhamnopyranosyl (1 → 6)-β-D-glucopyranoside. Compared with the fruits, the leaves are a richer source of phenolic compounds, such as phenolic acids. The phenolic acids estimated in the leaves by our team include: chlorogenic, p-coumaric, p-hydroxybenzoic, protocatechuic, salicylic, syringic acids (Szopa and Ekiert 2012), and also gentisic and ferulic acids (Mocan et al. 2014), and the precursor of one group of phenolic acids—cinnamic acid, was determined, too (Sovová et al. 2007).

### *Schisandra sphenanthera*

By reviewing the research conducted in recent years, one can observe a clear trend in the search for new structures, especially from the group of lignans, in other species of the genus Schisandra. Currently, approximately 150 lignan derivatives with the dibenzocyclooctadiene skeleton have been isolated from plants of the Schisandraceae family, and a wide variety of biological activities exhibited by these lignans have been uncovered (Ren et al. 2010).

The largest number of studies is concerned with the second, in terms of availability and prevalence in China, species of the genus Schisandra—*S. sphenathera*. They involve determinations of the composition of extracts not only from the fruits, but also stems and leaves, and even roots. Analyses of the chemical composition of the fruits of *S. sphenathera* have revealed considerable differences in their composition in comparison with the fruits of *S. chinensis*. The predominant dibenzocyclooctadiene lignans in the fruits are: epigomisin O, 6-O-benzoylgomisin, benzoylgomisin U methylgomisin, schisantherins A–D, deoxyschisandrin, pre-gomisin, gomisins: C, S, K3, J and U, myristargenol A, sphenanlignan, interiotherin B, schisantherins D and E, arisantetralone A (Chen et al. 2013b; Ren et al. 2010). In addition to these, other lignans from this group have been isolated and identified in the fruits of *S. sphenathera* in recent years, including one polyoxygenated C18-dibenzocyclooctadiene lignan, and also moreover one from the group of 4-aryltetralin lignans, and two from 2,3-dimethyl-1,4-diarylbutane lignans (Chen et al. 2013b; Ren et al. 2010). On the other hand, analyses of the composition of the stems of *S. sphenanthera* have proved the existence, in addition to those previously known, of: gomisins B, G and O, epigomisin O, schisantherin A and D, marlignan E and angeloylgomisin Q, and also new dibenzocyclooctadiene lignans—schisphenlignans A–E (Liang et al. 2013). Moreover, the roots of *S. sphenathera* have been used to isolate and identify butane-type lignans, besides the known ones, such as: schiglaucins A and B, epoxyzuonin, talaumidin, myristargenol A, and also eight previously unknown tetrahydrofuran lignans (Jiang et al. 2015a) (Table 3).

### Semisynthetic and synthetic derivatives of schisandra lignans

The search for new structures from the group of dibenzocyclooctadiene lignans includes not only isolation from plant material, but also attempts to obtain them by partial chemical synthesis. For example, new derivatives of schisantherin A and 6,7-secoschisantherol A have been obtained by semisynthesis. Semisynthetic schisantherin A derivatives showed antiproliferative activity on four human cancer cell lines and Id1 (an inhibitor of DNA binding protein) and estrogenic potency (Liu et al. 2013). So far, the most successful chemical synthesis has been the obtaining of a synthetic schisandrin C analogue known as DDB (diphenyl dimethyl bicarboxylate) (Xian Nong Tan Brand^®^)—currently the most potent antihepatotoxic substance, which has been used in the treatment of chronic viral hepatitis and chemical or drug induced hepatic damage (Opletal et al. 2004; Wang and Xu 2008). This compound is available in South-East Asian countries in commonly practised therapy: in South Korea as a drug named Hutecs (brand name—Bipheran), Daewoo (Lebicel), Pacific (Livital), Samjin (Peaceliva) and UDB—biphenyl dimethyl dicarboxylate and ursodeoxycholic acid (Pharmaking), whereas in Vietnam as Daewoong (Didcartuss), Bididi and Fortec (ICA Biotechnological & Pharmaceutical) and PharmaKing (Nissel) in Myanmar as Hepasel (Beijing Union).

Nevertheless, the world pharmaceutical market is dominated by preparations (drugs and food supplements) of natural origin, based on *S. chinensis* fruit extract. They are quite popular in USA and East Asia countries, but until now are less common in Europe.

## Main biological modes of action of dibenzocyclooctadiene lignans

Dibenzocyclooctadiene lignans are a large group of chemical compounds with different modes of action. It is, however, possible, to identify the main directions of their biological activity (Hancke et al. 1999; World Health Organization 2007), namely: blocking of calcium channels (Ca^2+^) (Chiu et al. 2008), reducing the level of: serum glutamic pyruvic transaminase (SGPT), liver glutamic-pyruvic transaminase (LGPT), alanine aminotransferase (ALT), aspartate aminotransferase (AST), inhibition of cyclooxygenase 1 and 2 (COX 1 and 2), inhibition of  the production of nitric oxide (NO) (Blunder et al. 2010; Hu et al. 2014), inhibition of gene expression of proinflammatory cytokines (Oh et al. 2010), and inactivation of cytochrome P450 (Wan et al. 2010).

In recent years, other valuable biological properties have been demonstrated for some dibenzocyclooctadiene lignans, such as: inhibition of platelet aggregation, the action of inhibiting the proliferation of human immunodeficiency virus (HIV) (Shi et al. 2014a) and adjuvant action in cancer treatment by: inhibiting early oncogenic activation of the Epstein-Barr virus (EBV-EA) (Chen et al. 2002), reversing multidrug resistance dependent on P-glycoprotein (Pgp-MDR) in tumor cells (Fong et al. 2007), and sensitization of liver cancer cells to the effects of doxorubicin (Hou et al. 2015; Li et al. 2006).

## Current knowledge of bioactivities and pharmacological applications

*Schisandra chinenisis* fruit is known as multifunction plant raw material. A lot of valuable applications of these species have been proved by scientific studies recently. Activities proved by scientific studies and pharmacological applications were described below and additionally were summarized in Table 4.

**Table 4:** Main biological activities of dibenzocyclooctadiene lignans

Lignan	Biological activity	References
Deoxyschisandrin	Anticancer, antiviral, hepatoprotective	Casarin et al. (2014), Jiang et al. (2015b), Xu et al. (2015)
Gomisin A	Anticancer, antiviral, anti-inflammatory, hepatoprotective	Jiang et al. (2015b), Waiwut et al. (2012)
Schisandrin	Antioxidative, hepatoprotecitve, sedative	Jeong et al. (2013)
Schisandrin B	Antioxidative, anticancer, antiviral, anti-inflammatory, hepatoprotective	Casarin et al. (2014), Chiu et al. (2006), Hu et al. (2014), Thandavarayan et al. (2015), Xu et al. (2015), Yim et al. (2009)
Schisandrin C	Antioxidative, anti-inflammatory, hepatoprotective	Jiang et al. (2015b)
γ-Schisandrin	Anticancer, antioxidative, hepatoprotective	Thandavarayan et al. (2015), Yim et al. (2009)
Schisanthenol	Antiviral	Xu et al. (2015)

### Hepatoprotective activity

Hepatoprotective activity is the best known profile of action of *S. chinensis* fruit extracts and of individual dibenzocyclooctadiene lignans. The available literature contains many reports on scientific studies that confirm these properties of the fruit extracts. The most recent ones concern detailed studies on the mode of action of individual lignans. For example, the mechanism of action of gomisin A has been studied. It has been shown that it increases the microsomal activity of: cytochrome B5, P450, NADPH cytochrome C reductase, N-demethylase aminophenazone, 7-ethoxycoumarin O-deethylase, and reduces the activity of 3,4-dibenzopyrene hydroxylase. It also accelerates the proliferation of hepatocytes, the endoplasmic reticulum, and the hepatic flow (Panossian and Wikman 2008; World Health Organization 2007).

On the other hand, it has been demonstrated that the mechanism of the hepatoprotective action of γ-schisandrin is based on increasing the concentration of mitochondrial glutathione. It has also been shown that γ-schisandrin raises the concentration of vitamin C in the liver in test animals, which may also have an impact on its protective effect on hepatocytes and oxidation of lipids. Moreover, schisandrin B has been shown to protect against oxidative damage to liver tissues, too (Thandavarayan et al. 2015).

A recently conducted study on hepatoprotective effects of six schisandra lignans: gomisin A, schisandrin, deoxyschisandrin, schisandrin B, schisandrin C, and schisantherin A, on acetaminophen-induced liver injury has found that these effects are partially associated with the inhibition of cytochrome-mediated bioactivation. The results of morphological and biochemical assessment of this study demonstrated the protective effects of all the tested lignans against acetaminophen-induced liver injury. Among the investigated oxidative damage in liver, heart and exerted the strongest hepatoprotective effects (Jiang et al. 2015b).

### Anti-inflammatory activity

The mechanism of anti-inflammatory action of schisandra lignans has been elucidated. It is based on the inhibition of the action of nitric oxide (NO) and the production of prostaglandins by stimulating the release of cyclooxygenase 2 (COX2) and inhibition of the expression of nitric oxide synthase (NOS) (Hu et al. 2014).

### Antioxidative and detoxification activities

Studies on the antioxidative and detoxification mechanisms of action of dibenzocyclooctadiene lignans have shown that they inhibit microsomal lipid peroxidation, reduce the concentration of superoxide radicals, inhibit microsomal NADPH oxidation in hepatocytes and reduce the release of alanine aminotransferase (ALT) and lactate dehydrogenase, which increases membrane integrity and viability of hepatocytes (Yim et al. 2009). Moreover, the mechanism of protective, antioxidant and detoxifying action of lignans on hepatocytes is based on the increase in hepatic glutathione levels, and the activity of glutathione reductase and glutathione S-transferase (Miao et al. 2009). The latest studies have shown that schisandrin B attenuates doxorubicin induced cardiac dysfunction via antioxidative and anti-inflammatory effects. Schisandrin B has been shown to protect against oxidative damage in liver, heart and brain tissues in rodents (Chiu et al. 2008; Thandavarayan et al. 2015).

### Anticancer activity

Anticancer activity of schisandra lignans has been studied in recent years. Researchers have been identifying the compounds responsible for this activity and studying its mechanisms. They have shown that gomisin A acts by inhibiting the production of the placental form of glutathione S-transferase (tumor marker) in hepatocytes and by increasing the excretion of the carcinogen; it also influences cytokinesis and reduces the number of focal neoplastic lesions in the liver. Other studies on gomisin A have shown anticancer activity on the colon carcinoma. It displayed apoptotic activity through caspase-7 cleavage in colon carcinoma HCT-116 cells (Hwang et al. 2011). A recent investigation concerned the effects of gomisin A on cancer cell proliferation and cell cycle arrest in HeLa cells. Gomisin A significantly inhibited cell proliferation, especially in the presence of tumour necrosis factor-α (TNF-α) (Waiwut et al. 2012).

In vitro studies on human leukemia cells—U973, it has been shown that another schisandra lignan—gomisin N, induces their apoptosis. The same mechanism of action of gomisin N has also been confirmed for hepatoma cells (Yim et al. 2009).

Apart from gomisin N, studies of the mechanism of anticancer action against two human tumor cell lines (adenocarcinoma cells—2008 and colon adenocarcinoma cells—LoVo) also included deoxyschisandrin. Both these lignans inhibited cell growth in a dose-dependent manner on both cell lines, but by inducing different types of cell death. In particular, deoxyschisandrin caused apoptosis in colon adenocarcinoma cells (LoVo) but not in ovarian adenocarcinoma cells (2008), while gomisin N induced apoptosis on both the cell lines used (Casarin et al. 2014).

As it turns out, the responsibility for the antitumor activity of the extracts from the fruits of *S. chinensis* is carried not only by dibenzocyclooctadiene lignans, but also by their polysaccharide fraction. A recent study confirmed the antitumor and immunomodulatory activities of water-soluble low-molecular-weight polysaccharide from *S. chinensis* (Zhao et al. 2013).

### Immunostimulant activity

Research conducted in recent years has focused on studying the biological activity of the polysaccharide fraction from extracts of the fruits of *S. chinensis*. The immunostimulatory, immunomodulatory and antitumor action of water-soluble low-molecular-weight polysaccharide from *S. chinensis* has been proved. The polysaccharide fraction exhibited immunomodulating properties, such as improving the weight of immune organs, enhancing the phagocytic activity of peritoneal macrophages, promoting hemolysin formation, and increasing lymphocyte transformation (Chen et al. 2012; Zhao et al. 2013).

### Influence on the central nervous system, adaptogenic and ergogenic activities

The dibenzocyclooctadiene lignans contained in the extract from the fruits of *S. chinensis* represent a group of natural chemical structures, which are now considered protectants against neuronal cell death and cognitive impairment in neurological disorders. In addition, the activity of polysaccharides contained in the fruits of *S. chinensis* is also studied in this respect. It has been shown that they, too, play an important role by raising the level of neurotransmitters in the central nervous system (Chen et al. 2012; Zhao et al. 2013).

Extracts from the fruits of *S. chinensis* are used in neurasthenia and states of exhaustion; they improve the ability to learn and memorize, indirectly increase alertness, improve concentration and mental performance. They are used as adjuvant substances in the treatment of Alzheimer’s, Parkinson’s, and Meniere’s diseases, and ADHD (attention deficit hyperactivity disorder). The fruit extracts exhibit antidepressant activity without inducing drowsiness (Jeong et al. 2013; Sa et al. 2015).

The fruits of *S. chinensis* have been recorded in some East Asian pharmacopoeias as an effective somnificant for the treatment of insomnia. However, the mechanism of the sedative and hypnotic effects of this plant raw material is still unclear. It has been shown that schisandrin—the dominant compound in *S. chinensis* fruit extract, produces beneficial sedative and hypnotic bioactivity, which might be mediated by the modification of the serotonergic system (Zhang et al. 2014a).

Moreover, adaptogenic and ergogenic properties of extracts from *S. chinensis* fruits were already well known from the traditional use of this plant. For this reason, *S. chinensis*, besides such plant species as *Eleutherococcus senticosus, Panax ginseng, Panax quinquefolius* or *Rhodiola rosea,* is classified as one of the worldwide known adaptogenic raw material (Winston and Maimes 2007).

### Influence on the respiratory system

The most recent studies of the pharmacological properties of *S. chinensis* fruit extracts have reported a beneficial role in the treatment of some respiratory system disorders.

Data from 2014 suggest that *S. chinensis* fruit extracts have possible application as an antiasthmatic drug because they lower airway hyperresponsiveness, immunoglobuline E level, and immune cell infiltration in mice with asthma. The results also suggest that the fruit extracts could be used as a complementary or alternative medicine to glucocorticoids (Kim et al. 2014a).

It has also been shown that extracts from the fruits of *S. chinensis* reduce cough frequency and pulmonary inflammation in cough hypersensitivity induced in guinea pigs by exposure to cigarette smoke. Lignans were indicated as the compounds likely to be responsible for this effect (Zhong et al. 2015).

### Influence on the cardiovascular system

Recently, *S. chinensis* fruit extracts and the dibenzocyclooctadiene lignans contained in them have gained attention for their potential role in the treatment of cardiovascular diseases, such as hypertension and myocardial infarction (Alexander and Wang 2012; Chun et al. 2014; Park et al. 2012a), which corroborates the observed effects of this species in traditional settings. Scientific investigations of the molecular mechanisms behind the observed phenomena have revealed that *S. chinensis* fruit extracts and its lignans exert cardiovascular protective activity by controlling multiple signalling pathways involved in various biological processes, such as vascular contractility, fibrosis, inflammation, oxidative stress, and apoptosis (Chen et al. 2013a; Chun et al. 2014).

### Anti-obesity activities

*S. chinensis* fruit extract has been evaluated for inhibition effects on adipocyte differentiation in 3T3-L1 cells and anti-obesity properties in induced obese rats. *S. chinensis* fruit extracts inhibited preadipocyte differentiation and adipogenesis in cultured cells, leading to decreased body weight and fat tissue mass in high-fat diet obese rats (Park et al. 2012b).

### Antiviral activity

The latest research has dealt with antiviral activity of dibenzocyclooctadiene lignans. Assays have shown that schisandrin B and deoxyschisandrin selectively inhibited the HIV-1 reverse transcriptase-associated DNA polymerase activity. Schisandrin B was able to impair the early phases of HIV-1 replication in cell-based assays. In addition, schisandrin B was also able to impair HIV-1 reverse transcriptase drug resistant mutants and the early phases of viral replication. Structure activity relationship revealed the importance of cyclooctadiene ring substituents for efficacy (Xu et al. 2015).

Schisandra lignans are also active against plant viruses. It has been demonstrated that the natural product—schisanthenol and its synthetic derivatives, were active against tobacco mosaic virus (Wang et al. 2015).

### Antibacterial activity

The essential oil, and also an extract from schisandra berry showed antibacterial effects against Gram positive (*Staphylococcus epidermidis, Staphylococcus aureus, Bacillus subtilis*) and Gram negative (*Chlamydia pneumoniae, C. trachomatis, Escherichia coli, Pseudomonas aeruginosa, Proteus vulgaris*) bacteria (Chen et al. 2011; Hakala et al. 2015).

## Use in cosmetics

Due to their numerous biological properties and rich chemical composition, extracts from *S. chinensis* fruit have a beneficial effect on the condition of the skin. Traditionally, they are attributed the following activities: toning, moisturizing, wound-healing, and narrowing dilated blood vessels. Therefore, they have found use in cosmetic preparations. Cosmetics containing extracts of fruit of *S. chinensis* purify and strengthen the protective barrier of the skin and soothe irritations. Extracts from *S. chinensis* fruit have also found use in hair care formulations. The extracts have anti-aging and revitalizing action, confirmed by sscientific research (Dweck and Marshall 2009; Quirin 2008). In addition, studies have shown that schisandrin B stimulates the production of glutathione, the body’s most potent natural antioxidant, which further confirms the validity of the use of extracts from the fruits of *S. chinensis* in anti-aging products (Chiu et al. 2011).

An extract from the fruit of *S. chinensis* was approved for use for cosmetic purposes and patented by BASF Beauty Care Solutions (Henry et al. 2012). In 2013, the company Laboratoires Expanscience patented, for the same purposes, an extract from the fruit of another species—*S. sphenathera* (Garnier and Msika 2013).

## Structure–activity relationships of lignans

There are only a few works which describe structure–activity relationships of lignans from *S*. *chinensis.* Currently researches are focused on describing of structure-anticancer activity of lignans. The presence of a hydroxyl group at the C7 position reduced or abolished such properties. The first effect is not surprising since the presence of the hydroxyl group results in an increase of the hydrophilicity, which decrease permeability into the lipid bilayer (Gnabrea et al. 2010). On the other hand increased activity was observed when a methylenedioxy group was present between C12 and C13. Anticancer action of lignans was also connected with P-glycoprotein inhibition. Some dibenzocyclooctadiene lignans possess three main structural features involved in P-glycoprotein inhibition: a 1,2,3-trimethoxy moiety, a 6-acyloxy group, and the absence of a 7-hydroxy group (Slanina et al. 2014). The most effective anticancer properties based on structure–activity relationships studies were shown for gomisin N and deoxyschisandrin (Gnabrea et al. 2010; Slanina et al. 2014).

The next experiments elaborating structure–activity relationships of lignans were focused on their action as platelet activating factor antagonists, where 6,7-dehydroschisandrol A showed the highest activity. Strong antiplatelet activity was shown for lignans without an ester group at C6, a hydroxyl group at C7, or a methylene dioxy moiety, and with an R-biphenyl configuration (Lee et al. 1999).

About antioxidant activity of schisandra lignans decided the exocyclic methylene group. It’s essential for this activity. The benzoyloxy group enhancing such effects (Choi et al. 2006).

## Analytical methods in determination of dibenzocyclooctadiene lignans

Because of described above applications of isolated lignans and extracts from schisandra fruit in pharmaceuticals, food supplements and cosmetics as well as interest in scientific research, many qualitative and quantitative analytical methods were elaborated. Among them the chromatographic methods prevail, including mainly high-performance liquid chromatography (HPLC) and thin layer chromatography (TLC) because of their good separation potential.

The current pharmacopoeial monographs indicate schisandrin determination as an assay. The reference technique used for standardization, mentioned by European Pharmacopoeia 8th (2013), Polish Pharmacopoeia X (2014) and WHO monograph (2007) is HPLC. An HPLC method was utilized in determination of schisandrin and gomisin A in schisandra fruit and leaves extracts, and in biomass extracts from in vitro cultures (Halstead et al. 2007; Lee and Kim 2010; Zhang et al. 2009).

The second technique frequently used in analysis of dibenzocyclooctadiene lignans in *S. chinensis* preparations is TLC. European Pharmacopoeia monograph recommend the use of TLC method to distinguish *S. chinensis* from *S. sphenanthera*, because fruit of the second species has different biosynthetic profile and is not considered as pharmacopoeial raw material. Lignans in *S. chinensis* fruit were analyzed by TLC with DART-MS (direct analysis in real time-mass spectrometry) detection, or with densitometric detection (Ohno et al. 2006; Wang et al. 1991).

Other chromatographic techniques are employed rarely. Schisandrin and gomisin A were determined in different parts of plants (e.g. seeds, fruits, leaves and shoots) by gas chromatography-mass spectrography analysis (GC–MS) (Schwarzinger and Kranawetter 2004) or in seeds by capillary electrochromatography (CEC) (Kvasničková et al. 2001) or micellar electrokinetic chromatography (MEKC) (Tian et al. 2005). CEC and MEKC are sometimes considered as electrophoretic techniques.

The newest methods applied to determine lignans are: ultra-performance liquid chromatography (UPLC) (Guo et al. 2012; Kim et al. 2014b; Sun et al. 2013b; Yan et al. 2014; Zhou et al. 2011) and ultra-fast liquid chromatography (UFLC) (Wei et al. 2013). Both techniques provide better possibilities than in HPLC but obtained in different ways. UPLC requires less time and less mobile phase. In columns smaller particles are used and the pressure could be very high, even up to 1200 athmospheres. These advantages frequently result in better resolution. UFLC is completely new technique in which instead of high pressure the system and column performance were improved.

Another advances in analytical procedures used in researches of schisandra preparations concern new techniques of extraction and purification of samples. Among others we can mention counter current chromatography (Chu et al. 2013; Li et al. 2014; Zhu et al. 2014b, 2015), extraction with supercritical CO_2_ (Zhu et al. 2014a) extraction with ultrasounds (Zhang et al. 2014c), extraction with microwaves (Ma et al. 2012b) and application of supercritical antisolvent precipitation (Huang et al. 2013).

Recently our team elaborated method of qualitative and quantitative analysis of schisandrin and gomisin A—HPTLC (high performance thin layer chromatography) with densitometric detection, which was successfully applied for determination of these compounds in food supplements, extracts from fruits and leaves becoming from natural source, as well as in biomass from in vitro cultures (Ekiert et al. 2013).

## Biotechnological studies

Plant in vitro cultures and possibilities of increasing the accumulation of secondary metabolites created by plant biotechnology are a well-known, alternative source of many valuable biologically active secondary metabolites used in the treatment of many diseases and also in cosmetology and food industry.

In order to obtain satisfactory, from a practical point of view, amounts of biologically active metabolites in in vitro plant cultures, a specific strategy is used (Ramawat and Mathur 2007). The elements of this strategy are, among others, the selection of cell lines, optimization of culture conditions (including selection of the basic composition of the culture medium, selection of plant growth regulators and their amounts in the media and types of cultures—agar cultures, agitated cultures, lighting conditions, temperature), obtaining cultures with a high degree of organogenesis (cultures of shoots, roots), making use of biotic or abiotic elicitors (stressors) and genetic transformation. Increasing the biomass production and the development of micropropagation protocols are also important, often key, strategies in biotechnology.

The growing interest in the medicinal values of *S. chinensis* fruits in different parts of the world and the growing number of works documenting the very valuable biological activities of fruit extracts as well as the individual dibenzocyclooctadiene lignans have caused a lot of interest in this species among teams conducting research in the field of plant biotechnology (Szopa and Ekiert 2014).

### Micropropagation protocols

Most of the biotechnological researches is dedicated to the development of micropropagation protocols for *S. chinensis*, mainly to somatic embryogenesis methods. In vitro cultures of *S. chinensis* have been the object of research aimed at developing highly effective micropropagation protocols with the use of different initial organs (zygotic embryos, cotyledonary leaves and hypocotyls of germinated zygotic embryos and female flower buds) (Smíšková et al. 2005; Kim et al. 2005; Chen et al. 2010; Yang et al. 2011; Sun et al. 2013a) (Table 5).

**Table 5:** Studies on micropropagation protocols of *S. chinensis* via somatic embryogenesis

Explant	Media	PGRs	References
Zygotic embryos from seeds	MS; Westvaco	2,4-D; BA; IBA; TDZ	Smíšková et al. (2005)
Zygotic embryos from seeds	MS; Merkle and Somer	2,4-D; BA; Zt	Kim et al. (2005)
Cotyledonary leaves and hypocotyls of germinated zygotic embryos	MS	BA; GA_3_	Chen et al. (2010)
Female flower buds	MS	2,4-D; GA_3_	Yang et al. (2011)
Zygotic embryos from seeds	MS	2,4-D; TDZ; Zt	Sun et al. (2013a)

*PGRs* - plant growth regulators, *2,4*-*D* - 2,4-dichlorophenoxyacetic acid, *BA* - 6-benzylaminopurine, *GA*_*3*_ - gibberellic acid, *IBA* - 3-indolebutyric acid, *NAA* - 1-naphthaleneacetic acid, *TDZ* - thidiazuron, *Zt* - zeatin

### Endogenic accumulation of bioactive secondary metabolites

Apart from micropropagation studies, research has been focused on another biotechnological aspect of in vitro cultivation of medicinal plant species—the endogenic accumulation of secondary metabolites in the biomass cultured in vitro. These studies seem to be the most important from a medicinal point of view. Under these experiments the main attention is focused on dibenzocyclooctadiene lignans in the biomass of in vitro cultures of *S. chinensis*. The biotechnological possibilities of producing some schisandra lignans have been documented by teams from the Czech Republic, Japan and Poland.

The first work of two cooperating teams from Masaryk University, in Brno, presents the results of only a qualitative analysis of three lignans: deoxyschisandrin, γ-schisandrin and schisantherin A (Havel et al. 2008).

A much broader scope of research is presented by the second work by scientific workers from Masaryk and Mendel Universities in Brno (Březinová et al. 2010). In their study different cell lines were examined; three lines of embryogenic callus in agar cultures were maintained in the dark (lines: LA-4, LAT-52, LA-58) and five lines of suspension cultures were maintained in a photoperiod (lines: LA-1, LA-4, LA-10, LAT-52, LA-58). All of the cell lines were grown on Westvaco medium-WV5 (Lloyd and McCown 1980), with 2 % sucrose, 2 mM glutamine and plant growth regulators (PGRs): 6-benzylaminopurine (BA) and 2,4-dichlorophenoxyacetic acid (2,4-D). In the embrogenic callus solid cultures there were six lignans present: schisandrin, gomisin A, deoxyschisandrin, γ-schisandrin, gomisin N and schisandrin C. In the suspension cultures of the five cell lines, the presence of all six compounds tested for was also revealed, although some of them were present only in trace amounts. As a result of the selection of the cell lines, only some of the compounds could be obtained in amounts that might be of interest from a practical point of view: in embriogenic callus solid cultures—gomisin N (54.7 mg/100 g DW (dry weight)), in suspension cultures—γ-schisandrin (55.0 mg/100 g DW), gomisin N (26.7 mg/100 g DW) and schisandrin C (20.9 mg/100 g DW) (Table 6). The most effective cell line in solid callus cultures was LAT-52, in suspension cultures LAT-52 and LA-1. The main compounds in the plant organs analyzed for comparative purposes were different lignans—in the seeds: schisandrin and deoxyschisandrin, while in the leaves: gomisin A and schisandrin.

**Table 6:** Maximal contents (mg/100 g DW) of dibenzocyclooctadiene lignans obtained by different research teams under biotechnological studies on in vitro cultures of *S. chinensis*

Lignans	Maximal content	Type of in vitro culture	Medium and PGRs	References
Angeoyl-/Tigloylgomisin H	26.66	Shoot-differentiating callus agar culture	MS BA—3 mg/l NAA—1 mg/l	Szopa et al. (2016a)
Angeoyl-/Tigloylgomisin Q	51.24	Shoot-differentiating callus culture	MS BA—3 mg/l NAA—1 mg/l	Szopa et al. (2016a)
Benzoylgomisin P	18.74	Stationary liquid shoot culture	MS BA—3 mg/l NAA—1 mg/l	Szopa et al. (2016a)
Deoxyschisandrin	308.51	Shoot-differentiating callus culture	MS BAP—3 mg/l NAA—1 mg/l	Szopa and Ekiert (2013)
Gomisin A	86.41	Shoot-differentiating callus culture	MS BAP—3 mg/l NAA—1 mg/l	Szopa and Ekiert (2011)
Gomisin F	40.00	Callus solid culture	½ MS KIN—0.05 mg/l 2,4-D—0.2 mg/l	Kohda et al. (2012)
Gomisin G	1.91	Shoot agitated culture	MS BA—3 mg/l NAA—1 mg/l	Szopa et al. (2016a)
Gomisin N	54.70	Embriogenic callus (cell line: LAT-52)	Westvaco BAP—0.9 mg/l 2,4-D—2.21 mg/l	Brezinová et al. (2010)
Schisandrin	70.54	Shoot-differentiating callus culture	MS BAP—3 mg/l NAA—1 mg/l	Szopa and Ekiert (2011)
Schisandrin B	20.70	Shoot agitated culture	MS BA—3 mg/l NAA—1 mg/l	Szopa et al. (2016a)
Schisandrin C	20.90	Suspension culture (cell line: LA-1)	Westvaco BAP—0.9 mg/l 2,4-D—2.21 mg/l	Brezinová et al. (2010)
γ-Schisandrin	55.00	Suspension culture (cell line: LA-1)	Westvaco BAP—0.9 mg/l 2,4-D—2.21 mg/l	Brezinová et al. (2010)
Schisanthenol	1.28	Shoot agitated culture	MS BA—3 mg/l NAA—1 mg/l	Szopa et al. (2016a)
Schisantherin A	2.97	Shoot agitated culture	MS BA—3 mg/l NAA—1 mg/l	Szopa et al. (2016a)
Schisantherin B	5.70	Shoot agitated culture	MS BA—3 mg/l NAA—1 mg/l	Szopa et al. (2016a)
Schisantherin D	5.65	Stationary liquid shoot culture	MS BA—3 mg/l NAA—1 mg/l	Szopa et al. (2016a)

Another work, which is a result of cooperation of three research units from Hiroshima University and Graduate School of Biomedical Sciences from Hiroshima, presents the possibility of producing two of the six lignans analyzed—gomisin A and gomisin F, in the established callus cultures. Callus cultures were maintained on Murashige and Skoog (MS) (1962) and Woody Plant (WP) (Lloyd and McCown 1980) media in full and half strength (1/2 MS or 1/2 WP) salt formulations, additionally following PGRs were tested: 2,4-D, BA, 3-indolebutyric acid (IBA) and kinetin (KIN). All biomass extracts contained only two dibenzocyclooctadiene lignans: gomisin A and F. Under the chosen optimal conditions, gomisin A and gomisin F contents were 0.05 and 0.04 g % DW, respectively. Interestingly, they were higher than in the plant organs (leaves and fruits) analyzed for comparative purposes (Kohda et al. 2012) (Table 6).

Our team from the Jagiellonian University, Kraków, has been conducting studies on in vitro cultures of *S. chinensis* since 2010. These studies seem to be the most advanced in relation to the accumulation of dibenzocyclooctadiene lignans. Established in vitro cultures were used to perform studies concerning the accumulation of dibenzocyclooctadiene lignans in the biomass of *S. chinensis* in vitro cultures at different stages of morphogenesis: shoot-differentiated callus and undifferentiated callus cultures, and also in different forms: an agar, stationary liquid and agitated cultures. Cultures were tested in different conditions; different media: MS and Linsmaier and Skoog (LS) (1965), and various concentrations of PGRs: BA and 1-naphthaleneacetic acid (NAA), were tested. Apart from schisandra lignans, qualification and quantification of phenolic acids were also performed. Under the research, almost all of the biotechnological steps of the cultivation of plants in vitro cultures were completed.

At the beginning, after the initiation of cultures, the impact of different plant growth regulators: BA and NAA, on the accumulation of schisandra lignans was studied. Extracts from the biomass of shoot-differentiating and undifferentiating callus cultures of *S. chinensis* growing on variants of the MS medium, with different concentrations of BA and NAA were analyzed for the accumulation of six lignans: schisandrin (max. 70.54 mg/100 g DW) and gomisin A (max. 86.41 mg/100 g DW) (Szopa and Ekiert 2011), deoxyschisandrin (max. 308.51 mg/100 g DW) and γ-schisandrin (max. 22.09 mg/100 g DW) (Szopa and Ekiert 2013) and gomisin G (max. 21.89 mg/100 g DW) and schisantherin A (max. 33.45 mg/100 g DW) (Szopa and Ekiert 2015) (Table 6). The amounts of these compounds in the biomass extracts were dependent on the concentration of plant growth regulators in the MS medium. The amounts of the tested compounds found in the extracts from the undifferentiated callus cultures were considerably smaller than in the extracts from cultures with a higher degree of differentiation. In addition, the identity of the three lignans estimated (deoxyschisandrin, gomisin A and schisandrin), after isolation and purification by chromatographic methods from the biomass cultured in vitro, was also confirmed by spectral analysis (^1^H-NMR) (Szopa et al. 2015).

Under the latest studies, performed 3 years after the previous experiments, in cooperation with a teams from Medical University of Gdańsk and Nicolaus Copernicus University of Toruń shoot-differentiating callus cultures of *S. chinensis* were cultured in different in vitro systems—in an agar system and also in two different liquid systems: stationary and agitated. Quantification of fourteen dibenzocyclooctadiene lignans (angeoyl-/tigloylgomisins H and Q, benzoylgomisin P, deoxyschisandrin, gomisins A and G, schisandrin, schisandrins B and C, γ-schisandrin, schisanthenol, schisantherins A, B and D) was performed in the extracts from the biomass and from the media. Some of them (angeoyl-/tigloylgomisins H and Q, benzoylgomisin P, schisantherins D), were tentatively identified. The main lignans detected in the biomass extracts from all the tested systems were: schisandrin (max. 65.62 mg/100 g DW), angeloyl-/tigloylgomisin Q (max. 49.73 mg/100 g DW), deoxyschisandrin (max. 43.65 mg/100 g DW) and gomisin A (max. 34.36 mg/100 g DW) (Table 6). The lignans were not detected in the media. The amount of the main schisandra lignan—schisandrin, was 2.21 times higher in biomass extracts than in the extracts from the leaves of the parent plant, analyzed for comparison, but 2.02 times lower than in the fruits. Also, the maximum amounts of the other main lignans: gomisin A and deoxyschisandrin, were comparable with their amounts in the leaves, but 3.18 and 1.39 times lower than in the fruits, respectively (Szopa et al. 2016a).

High amounts of lignans are also determined by the high degree of organogenesis of the biomass studied. These results suggest that the in vitro culture of *S. chinensis* can be a potential alternative source of lignans. The other significant outcome of the studies performed so far is the finding that the investigated biomass is suitable for cultivation in liquid systems; therefore, *S. chinensis* in vitro cultures are a good model for further studies on the accumulation of lignans, particularly in large-scale installations (bioreactors). These studies are in progress. Experiments are being performed on the basis of large-scale bioreactors such as: a bubble column with shoot immobilization system, balloon-type bubble and spray bioreactors, and also some commercially available constructions.

A comparative list of the maximum amounts of dibenzocyclooctadiene lignans obtained by different research teams under biotechnological studies in various types of in vitro cultures of *S. chinensis* is shown in Table 6.

A study focused on the analysis of phenolic acids in the biomass of in vitro cultures of *S. chinensis* was also performed by our team (Szopa and Ekiert 2012). The amounts of free phenolic acids and cinnamic acid were determined using the HPLC method in extracts from the biomass of *S. chinensis* at different stages of organogenesis (shoot-differentiating and undifferentiating callus cultures), cultured in vitro on several variants of the MS medium, containing different concentrations of PGRs, and in extracts from overground parts of plants growing in vivo. The acids detected in the extracts from in vitro cultured biomass included: chlorogenic, p-coumaric, p-hydroxybenzoic, protocatechuic, salicylic and syringic acids. The maximum total amounts of the metabolites estimated in the biomass of shoot-differentiating callus culture was equal to 60.05 mg/100 g DW. Interestingly, the maximum total content in biomass extracts from undifferentiating callus culture, with a lower degree of differentiation, was higher and amounted to 78.24 mg/100 g DW. The maximum total amounts of phenolic acids in both types of in vitro cultures were greater than in the fruits and leaves of plants growing in vivo. The studies on the accumulation of phenolic acids in the biomass cultivated in different bioreactors are also in progress.

### Biotransformation reactions

**Fig. 4:**
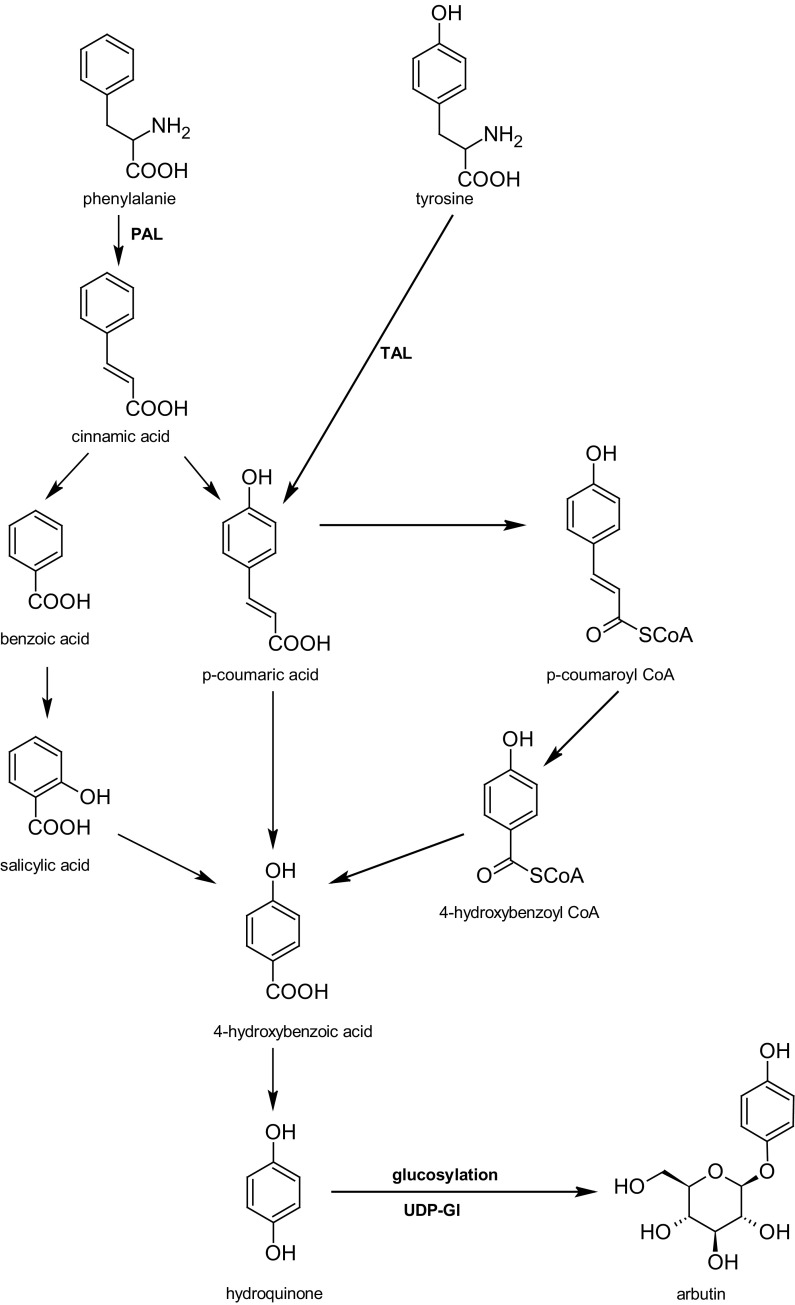
Possible ways of arbutin biogenesis. *PAL* - phenylalanine ammonia-lyase, *TAL* - tyrosine ammonia lyase, *UDP*-*Gl* - uridine diphosphate glucose

A quite interesting aspect of the biotechnological studies that were performed on in vitro cultures of *S. chinensis* was the testing of the biotransformation capability of the cells cultured in vitro. These studies use the enzymatic potential of plant cells cultured in vitro for the biotransformation of exogenously supplied simple substrates to expected products. The most notable reaction is O-β-D-glucosylation of hydroquinone into arbutin, an important product in therapy and cosmetology. The possibility of using microorganisms in these processes is limited because the reaction of O-α-D-glucosylation proceeds in microorganisms better than O-β-D-glucosylation (Fig. 4).

Preliminary studies on biotransformations were performed by a team from Charles University in Prague. The researchers studied the biotransformation capacity of various exogenous precursors of arbutin and salicin (phenylalanine, salicylic aldehyde, hydroquinone, cinnamic acid, o-coumaric acid, p-coumaric acid, p-anisoic acid, helicin and salicylic acid) in in vitro cultures of four plant species, including *S. chinensis* callus cultures. In *S. chinensis* in vitro cultures there occurred β-D-glucosylation of hydroquinone into arbutin. The maximum amount of the product, equal to 5.08 g %, was obtained 7 days after administering the precursor at a dose of 100 ml per litre of medium (Dušková et al. 1999; Dušková et al. 2005).

**Fig. 5:**
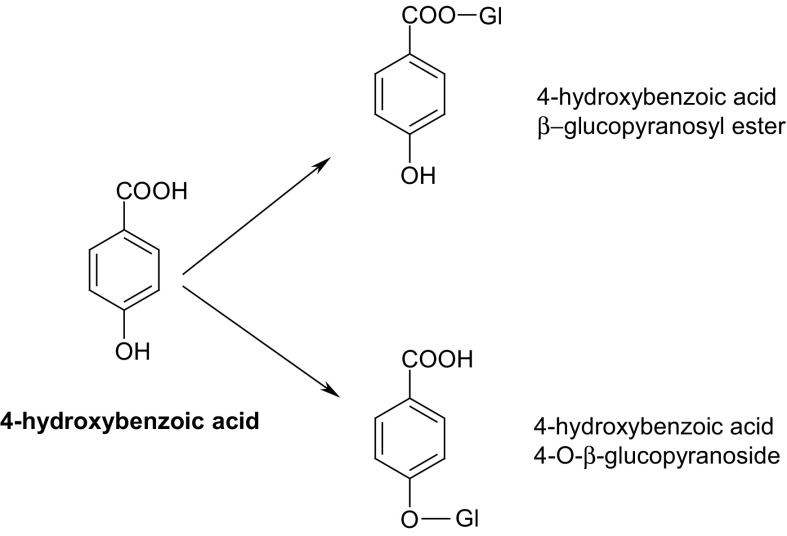
4-Hydroxybenzoic acid biotransformation products

In the latest research, an experiment on the enzymatic potential of cells from in vitro culture of *S. chinensis* in biotransformation processes was also undertaken by our team from Jagiellonian University, Kraków. Under this study, optimization of the process of biotransformation of hydroquinone into arbutin was performed in agitated shoot cultures of this plant species. The optimization involved testing various concentrations of the precursor (100–400 mg/l of medium) and different ways of administering it (single dose, or divided into two or three portions). Arbutin was accumulated mainly in the in vitro cultured biomass (85.2–98.6 %); the highest amount (39.02 mg/g DW) was found after administering of 384 mg/l hydroquinone in a dose divided into two portions. A preliminary experiment with the biotransformation of 4-hydroxybenzoic acid as a precursor did not produce any arbutin but a mixture of two other compounds, products of glucosylation of the precursor—4-hydroxybenzoic acid 4-O-β-glucopyranoside and 4-hydroxybenzoic acid β-glucopyranosyl ester (Fig. 5). The identity of all the biotransformation products was confirmed by ^1^H-NMR analysis. These results additionally indicate a widespread possibility of the enzymatic glycosylation reactions in in vitro plant cultures (Szopa et al. 2016b).

## Conclusions

Presented review proved, that *S. chinensis* is very plausible object of the different scientific studies. Chemical analyses have been concerned with the isolation and identification of new structures of secondary metabolites occurring in the fruits and also in the leaves and stems of *S. chinensis*, and in other Schisandra species. The current pharmacological researches confirm the already known activities of *S. chinensis* and report on new potential health-promoting activities of this species and its usefulness also in cosmetology. The studies have been focused on fruit extracts and also on the activities of individual dibenzocyclooctadiene lignans. The developed analytical methods, which are the best tool for qualification and quantification assays of schisandra lignans, are also becoming increasingly more precise, also important are elaborated new techniques of extraction and purification of samples. Moreover, the phytochemical studies are being expanded and enriched with biotechnological studies. The biotechnological methods of producing dibenzocyclooctadiene lignans (especially micropropagarion and endogenic accumulation) that are so valuable from a pharmacological point of view seem to provide a very promising alternative way of obtaining these metabolites in the future.
